# Pituitary Remodeling Throughout Life: Are Resident Stem Cells Involved?

**DOI:** 10.3389/fendo.2020.604519

**Published:** 2021-01-29

**Authors:** Emma Laporte, Annelies Vennekens, Hugo Vankelecom

**Affiliations:** Laboratory of Tissue Plasticity in Health and Disease, Cluster of Stem Cell and Developmental Biology, Department of Development and Regeneration, KU Leuven (University of Leuven), Leuven, Belgium

**Keywords:** pituitary, plasticity, stem cells, organoids, regeneration, maturation, aging

## Abstract

The pituitary gland has the primordial ability to dynamically adapt its cell composition to changing hormonal needs of the organism throughout life. During the first weeks after birth, an impressive growth and maturation phase is occurring in the gland during which the distinct hormonal cell populations expand. During pubertal growth and development, growth hormone (GH) levels need to peak which requires an adaptive enterprise in the GH-producing somatotrope population. At aging, pituitary function wanes which is associated with organismal decay including the somatopause in which GH levels drop. In addition to these key time points of life, the pituitary’s endocrine cell landscape plastically adapts during specific (patho-)physiological conditions such as lactation (need for PRL) and stress (engagement of ACTH). Particular resilience is witnessed after physical injury in the (murine) gland, culminating in regeneration of destroyed cell populations. In many other tissues, adaptive and regenerative processes involve the local stem cells. Over the last 15 years, evidence has accumulated that the pituitary gland houses a resident stem cell compartment. Recent studies propose their involvement in at least some of the cell remodeling processes that occur in the postnatal pituitary but support is still fragmentary and not unequivocal. Many questions remain unsolved such as whether the stem cells are key players in the vivid neonatal growth phase and whether the decline in pituitary function at old age is associated with decreased stem cell fitness. Furthermore, the underlying molecular mechanisms of pituitary plasticity, in particular the stem cell-linked ones, are still largely unknown. Pituitary research heavily relies on transgenic *in vivo* mouse models. While having proven their value, answers to pituitary stem cell-focused questions may more diligently come from a novel powerful *in vitro* research model, termed organoids, which grow from pituitary stem cells and recapitulate stem cell phenotype and activation status. In this review, we describe pituitary plasticity conditions and summarize what is known on the involvement and phenotype of pituitary stem cells during these pituitary remodeling events.

## Introduction

One of the defining characteristics of the pituitary gland is its ability to plastically adapt its cell composition to fulfill changing endocrine demands of the body throughout life. The gland occupies a central position in the endocrine system, upstream receiving regulatory signals from the hypothalamus and downstream sending hormonal messages to endocrine organs throughout the body (such as adrenal and thyroid glands, ovaries and testes), thereby regulating the production of specific hormones by these target glands. Tight control of pituitary hormonal release is maintained *via* a strict balance between cues from the hypothalamus and negative feedback loops from the peripheral target hormones. The major endocrine part of the gland (i.e., anterior pituitary, AP) contains five endocrine cell types, each dedicated to produce (a) specific hormone(s). Somatotropes synthesize and secrete growth hormone (GH), generally involved in bone and organ growth and regeneration; lactotropes produce prolactin (PRL), playing an essential role in pregnancy and lactation; gonadotropes generate follicle stimulating hormone (FSH) and luteinizing hormone (LH), controlling fertility and reproduction; adrenocorticotropic hormone (ACTH) is produced by corticotropes and necessary in stress and immune responses; and thyrotropes make thyroid-stimulating hormone (TSH) which is indispensable in metabolism control (1, 2). Apart from these endocrine cells, the AP also houses non-hormonal cell types encompassing endothelial, immune, and folliculostellate (FS) cells. Existence of stem cells in the pituitary gland was theorized for many decades until their convincing disclosure 15 years ago and thorough description since then, along with the identification of a number of stem cell markers, positioning SOX2 at the head of the list (3–7). From the multiple studies set out to unveil the biological significance of this stem cell population, it is at present perceived that these cells, at least in the basal adult gland, are highly quiescent. Thorough insight into their function(s) is still not firmly achieved (2, 8).

During postnatal life, several physiological processes ask for adaptations in hormone balances and thus pituitary output. The gland shows the essential flexibility to alter hormonal production by remodeling its function and cellular composition in these conditions. For onset and development of puberty, GH and gonadotropins (LH and FSH) are needed to drive and regulate pubertal growth spurt and gonad maturation (through the sex steroid hormones testosterone and estradiol), respectively (9). Increased levels of PRL are needed during pregnancy and lactation [to enlarge and prepare mammary glands for milk production (10–12)], and elevated ACTH concentrations are necessary to cope with stress (13–15). Pituitary cell remodeling is also seen in early-postnatal life, when the (rodent) pituitary gland undergoes prominent growth and maturation (2, 16, 17). In contrast, remodeling capacity may be compromised at aging concurrent with pituitary functional decline (18). Finally, injury in the gland during postnatal life triggers a local regenerative remodeling process culminating in regeneration of tissue cells and hormonal function (19–22). In general, it is only poorly understood how the specific cellular changes during these remodeling events are brought about, and whether and how pituitary stem cells are involved.

In this review, we summarize dynamic adaptations in the pituitary cell landscape at key time points of postnatal life and during specific (patho-)physiological processes, and discuss the current knowledge regarding involvement of the resident stem cells in these remodeling processes. To gain deeper insight into stem cell phenotype and role, appropriate, malleable and reliable research models are indispensable. Therefore, we also give an overview of *in vitro* pituitary (stem cell) study models and the important improvements that have recently been achieved in this field, in particular by establishing organoids.

## Pituitary Stem Cells During Key Physiological Events of Postnatal Life

### Pituitary Stem Cells During Neonatal Maturation

When born, although all hormonal cell types are specified in the pituitary, the gland still needs to further expand and mature (23, 24). Before birth, during embryonic development [extensively reviewed elsewhere (25–27)], the pituitary initially appears as a thickening and subsequent invagination of the oral ectoderm called Rathke’s pouch (RP), occurring in the mouse at embryonic day (E)8.5. From this first recognizable “pituitary” structure, the AP and intermediate lobe [containing the melanocyte stimulating hormone (MSH)-producing melanotropes] eventually develop. Around E12.5, the pouch disconnects from the oral roof and forms a closed entity around a central lumen (the future cleft). The marginal zone (MZ) around this lumen houses the progenitor cells of the embryonically developing pituitary which proliferate and then start to colonize the nascent AP where they generate three main lineages from which the different hormonal cell types develop, i.e., the PIT1 lineage giving rise to somatotropes, lactotropes and thyrotropes; the SF1/GATA2 lineage from which the gonadotropes develop; and the TBX19 lineage turning into corticotropes (25, 26, 28). This genesis of cell lineages and cell types is steered by a specific interplay between evolutionarily conserved signaling factors, such as bone morphogenetic proteins (BMP) and fibroblast growth factors (FGF), and sonic hedgehog (SHH), wingless-type MMTV integration site (WNT) and NOTCH family members, resulting in tightly regulated spatiotemporal expression of transcriptional regulators which govern the development of the distinct cell types (25, 26, 29, 30).

During the first postnatal weeks, the rodent pituitary almost doubles in size. This expansion and maturation process is driven by increased cell proliferation [including the re-entry of embryonically committed cells into the cell cycle (31)] and expansion of cell size because of endocrine differentiation with accumulation of hormone-containing secretory granules. In the rat, the proportion of proliferating cells in the postnatal pituitary is highest in the first week after birth to subsequently decline in the coming weeks toward very low levels at adulthood [i.e., from 400 dividing cells/mm^2^ in the first week to 50 dividing cells/mm^2^ at 8 weeks of age (17, 32)]. The basal adult pituitary indeed shows low turnover, with hormonal cells being replaced only every 60–70 days [as estimated in the young-adult rat (33, 34)]. Proliferation during neonatal maturation is observed not only in freshly differentiated, granular hormone-producing cells, representing 70–80% of the dividing population (32, 35–37), but also in non-hormonal agranular cells, at least partly identified as FS cells based on morphology and expression of the FS cell marker S100 (17, 38, 39). Nowadays, it is known that the heterogenous FS cell population encompasses pituitary stem cells (4, 5, 40).

Following the discovery of pituitary stem cells, several findings were reported that point to their potential involvement in neonatal pituitary growth and maturation. First, the proportion of stem cells is highest during the first postnatal week [i.e., postnatal day (P)1-7 *versus* P21, marking the end of the growth wave in mice, and *versus* adult age], as explored by both functional “side population” (SP) phenotype [a protective efflux capacity of stem cells (3, 4)] and molecular SOX2^+^ nature (16). The topography of SOX2^+^ cells at birth shows multicellular layers in the MZ, particularly prominent in the merging region of the anterior and intermediate lobe (referred to as the wedges) where “streams” of SOX2^+^ stem cells appear to move into the developing AP (16). Moreover, the stem cell compartment shows a higher activation status involving a larger proportion of proliferating SOX2^+^ cells. In accordance, neonatal pituitary stem cells show increased sphere formation capacity, further demonstrating their intensified functionality. Spheres (referred to as pituispheres) have been shown to develop from pituitary stem cells and sphere formation capacity is used as a readout of stem cell functionality and activation (3–5) (see further below). Additional support for the activated phenotype of neonatal pituitary stem cells includes their enhanced expression of stemness and embryogenesis-related genes (e.g., *Sox2, Sox9, Prop1* and components of NOTCH, WNT and SHH pathways), and swifter differentiation into hormonal cells in pituisphere culture as compared to the adult pituitary stem cells (16). PROP1 is a transcription factor formerly considered only essential for the development of the PIT1-dependent cell lineages during pituitary embryonic development but nowadays endowed with a more general function in embryonic stem/progenitor cells, governing their migration from the MZ to the nascent AP (41). It was shown *in vivo* (using mutant mice) and *in vitro* (using stem cell-derived colony culture; see below) that PROP1 drives the stem/progenitor cells into epithelial-mesenchymal transition (EMT) which is considered to underlie their migratory movement toward the developing AP (42). PROP1 disappears during this transition from stem/progenitor cell state towards committed endocrine cell (41). The factor remains expressed in SOX2^+^ cells in the (rat) MZ acutely after birth, but the number of PROP1^+^/SOX2^+^ cells decreases during further postnatal maturation, with PROP1-expressing cells becoming rare in the adult pituitary (43), although this issue is still not fully settled since another study showed persistent expression (40). Also, NESTIN, a classic neural stem cell marker, has been identified in pituitary stem cells (3–5, 44). The proportion of NESTIN^+^ cells rapidly decreases after birth [from 12% just before birth to 2% in the adult gland, as determined in rat (45)], whereas some of these cells remain proliferating which has led to the proposal that they represent the small proliferative fraction of the pituitary stem cells. The majority (70–80%) of the neonatal NESTIN^+^ cells co-express PIT1 as well as dependent hormones (especially GH and PRL), suggesting that new hormonal cells might be differentiating from these NESTIN^+^ progenitor cells (45). PROP1^+^ and NESTIN^+^ stem/progenitor cells thus also appear to be involved in the neonatal growth and maturation phase of the (rat) pituitary gland, both strongly fading in abundance at later age.

**Figure 1:**
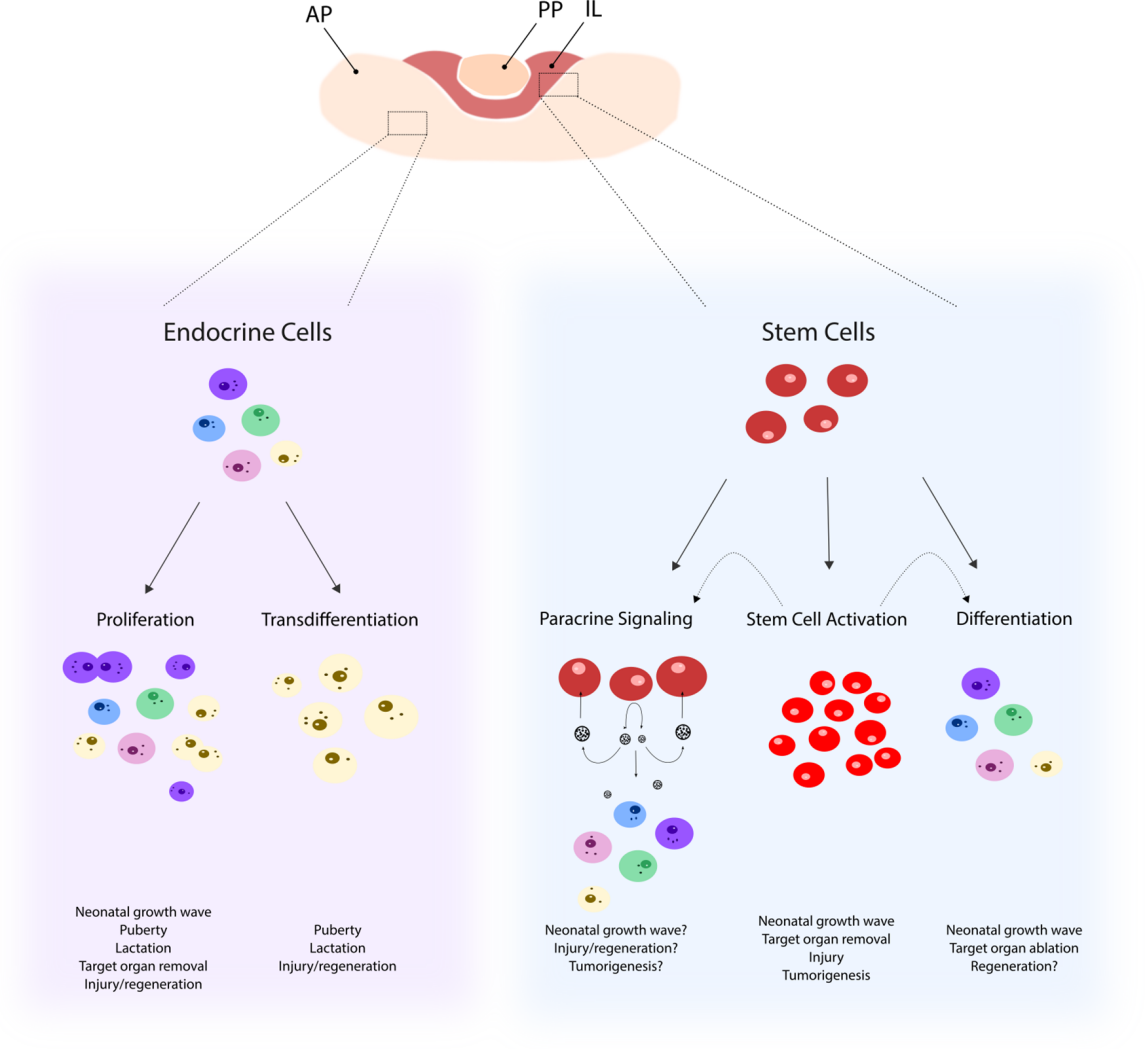
Possible mechanisms underlying remodeling of the pituitary cell landscape. Pituitary remodeling during postnatal (patho-)physiological processes may involve differentiated endocrine cells that either proliferate (as, for instance, observed in the neonatal growth wave) or transdifferentiate (as, for instance, seen during puberty). In addition, the stem cell compartment may become activated (involving increased proliferation and upregulated stemness factors and signaling pathways) as, for instance, observed following pituitary injury. Pituitary stem cells, activated or not, may contribute to new endocrine cell formation during the pituitary remodeling events through differentiation (as, for instance, after target organ removal such as adrenalectomy) and/or through sending paracrine signals to surrounding cells (as, for instance, likely to occur during tumorigenesis). AP, anterior pituitary; PP, posterior pituitary, IL, intermediate lobe.

Together, higher proportion and activation status of the resident stem cells suggest a role in neonatal development of the pituitary. Possible mechanisms include direct generation of new endocrine cells and/or paracrine stimulation of committed progenitor cells to proliferate and differentiate or of differentiated hormonal cells to proliferate and expand in number (**Figure 1**). The first hypothesis is supported by the finding of swifter differentiation pace of neonatal stem cells in pituisphere culture (16) and of more significant contribution of stem cells (SOX2^+^ or SOX9^+^) to hormonal cells when transgenic lineage tracing is started in newborns (and analyzed 4 weeks later) *versus* in adulthood (6). Intriguingly, the largest contribution of traced stem cells was found in the gonadotrope population (∼35%), which was proposed to be due to the use of tamoxifen to induce the lineage tracing, a selective estrogen receptor modulator which may affect gonadotrope development (6). Taken together, a clear view on degree and type of participation of stem cells in the neonatal phase of pituitary growth is not yet sketched. Further intriguingly, ablation of SOX2^+^ stem cells in the neonatal pituitary does not affect postnatal hormonal cell population development toward adult age (46). Increased proliferation in the remaining SOX2^+^ cells acutely upon the ablation process may account for this rescue of hormonal cell development in the maturing gland. Alternatively, the ablation grade may have been too low (30%) to provoke an effect in eventual hormonal cell population evolution (46). Fascinatingly, the stem cell population itself did also not restore to normal (as analyzed 4–6 months later) (46) which might indicate a redundancy of stem cells for normal postnatal pituitary development. Of note, double SOX2^+^/hormone^+^ cells are not detected during neonatal maturation (neither in the basal adult gland), which may indicate that stem cells first need to downregulate or shut off SOX2 expression before hormone expression can be initiated (16).

At present, it is not clear which molecular mechanisms or regulatory networks underlie the activation status of the neonatal pituitary stem cell compartment [in detail reviewed elsewhere (30)]. Deletion of the NOTCH downstream effector *RbpJ* in embryonic *Prop1^+^* progenitor cells leads to a significant decrease in proliferating SOX2^+^ cells in neonatal pituitary (from 30 to 15%), and almost complete absence of early-postnatal SOX2^+^ cells (31). Moreover, re-entry of embryonically committed PIT1^+^ cells into the cell cycle, as normally occurring shortly after birth, is drastically reduced. At adult age, the pituitary of these mice is hypoplastic with a clear decrease in all endocrine cell types thereby supporting an important role for NOTCH signaling in early-postnatal growth and maturation of the pituitary toward its adult form through governing both the SOX2^+^ cell self-renewal, maintenance and proliferation and the process and timing of PIT1^+^ cell re-entry into the cell cycle (31). Genetic deletion of *Notch2*, one of the NOTCH receptors expressed in the pituitary stem/progenitor cells during development and later in life (47, 48), as well as postnatal inhibition of NOTCH activity using the γ-secretase inhibitor DAPT, resulted in a similar phenotype, with decreased proliferation of the stem/progenitor cells and declined expression of the stem cell markers *Sox2*, *Sox9* and Grainyhead-like 2 (*Grhl2*), a transcription factor found to be specifically expressed in the pituitary stem cells of the embryonic and early-postnatal gland (49, 50). In agreement, it has been shown that the NOTCH target gene *Hes1* is indispensable in the correct control of progenitor cell expansion, since pituitaries lacking *Hes1* exhibit decreased proliferation and increased cell cycle exit [with higher expression of cyclin-dependent kinase inhibitors such as p27 and p57 (51, 52)]. Furthermore, *Prop1* is a direct target of NOTCH signaling, thereby also assigning a role to PROP1 in the robust PIT1 lineage hormone cell expansion after birth (49).

Taken together, pituitary stem cells display an activated phenotype during the neonatal growth and maturation process of the gland. Despite emerging data and views, further research is needed to pinpoint their actual position in this active molding process, i.e., to decipher whether it involves direct contribution or stimulation of existing cells, or both, and to define the exact molecular regulatory mechanisms responsible for their activated status and function (**Figure 1**).

### Pituitary Stem Cells During Puberty

Puberty encompasses sexual maturation of body and gonads, and growth of skeleton, bones, and organs. This key developmental process is essentially steered by hormones, in particular the gonadotropins LH and FSH (further mediated by their downstream effectors estradiol and testosterone), and GH [further mediated by liver-derived insulin-like growth factor-1 (IGF-1)] which is responsible for the pubertal growth spurt (9, 53, 54). The hypothalamic-pituitary-gonadal axis is already created and active in pre- and postnatal development. Its activity transiently declines during childhood (so-called “juvenile pause”) but is re-activated at the onset of puberty, signaling the start of pubertal development (55).

Surprisingly little is known about cell adaptations in the pituitary gland during this key phase of life. In mice, somatotropes and lactotropes reach their full cell size at the onset of puberty and ultrastructural features become similar to their counterparts in adult gland. Other (non-)endocrine cell types reach final cell size and morphology about one week after puberty (56). The increasing levels of GH coincide with a peak in somatotrope markers (e.g., *Pou1f1*, the gene encoding PIT1*)*, although it is not clear yet whether there is higher expression of GH per somatotrope cell or whether somatotrope number expands (40). A study in children reaching puberty showed that rising GH levels are due to a rise in amplitude of the GH secretion peaks, not to an increase in pulse frequency (57). Whether pituitary stem cells are involved in remodeling of somatotropes during puberty, is not yet known.

Gonadotropes also change during puberty in number and activity. In medaka fish (*Oryzias latipes*), FSH- and LH-producing cells show hypertrophy and estradiol-driven proliferation during puberty (58). One study related to the involvement of stem/progenitor cells showed that genetically sustained *Prop1* overexpression in gonadotropes results in delayed puberty as marked by decreased gonadotrope differentiation (with nearly absent *Lhb* and *Fshb* expression), less developed seminal vesicles in male mice and delayed vaginal opening, smaller ovaries and thinner uteri in female mice, as well as belated growth (59, 60). On the other hand, also knock-out (KO) of the *Prop1* gene results in underdeveloped gonads (61). Human patients with *PROP1* mutations either do not enter or complete pubertal development, show growth failure and need sex hormone substitutions (62). Taken together, PROP1^+^ pituitary stem/progenitor cells might play a role in normal pubertal development and the needed pituitary gonadotrope and somatotrope cell remodeling (**Figure 1**), but hard, convincing evidence is not available yet.

### Pituitary Stem Cells During Pregnancy and Lactation

Successful pregnancy and lactation largely depend on the action of, and regulation by PRL. Accordingly, PRL levels are substantially increased during these physiological states and processes (10–12). Whether this rise is substantially due to increasing numbers of lactotropes in the pituitary and if so, how new lactotropes emerge (i.e., proliferation of existing PRL-producing cells, transdifferentiation from somatotropes or differentiation from stem/progenitor cells), is still not unambiguously underpinned (**Figure 1**).

In rats, a peak in proliferating lactotropes is observed in late pregnancy (day 19–21 of gestation) resulting in an augmented proportion of PRL^+^ cells during lactation when proliferative activity already returned to the (low) baseline level (63, 64). In humans, lactotrope hyperplasia occurs in various phases of pregnancy, assigned to both proliferation of pre-existing PRL^+^ cells and transdifferentiation of somatotropes to lactosomatotropes [i.e., cells expressing both PRL and GH (65)]. In mice, no increase in lactotrope cell number was observed during lactation, but cell size of individual lactotropes was enlarged and cells formed long-lasting networks that enabled cell-cell communication and functional connectivity to coordinate elevated PRL secretion (10). Using PRL^eYFP/+^ reporter mice, a 20% volume increase of the “enhanced yellow fluorescent protein”-positive (eYFP^+^) cell population was found during lactation, but this study did also not observe a rise in the proportion of PRL^+^ cells (66). Still, proliferation may have occurred in the lactotrope progenitor cell population, a possibility supported by another study that also did not perceive an increase in proliferating PRL^+^ cells (neither in SOX2^+^ stem cells) during lactation, but detected a large surge in proliferating PIT1^+^ progenitor cells (31). Transdifferentiation between PRL^eYFP/+^ and GH^+^ cells was found to be low (less than 1%), excluding a major contribution of such conversion process during lactation in the mouse (66). Since a large volume increase of the pituitary (peaking at mid-lactation) was seen, which could not fully be explained by the increased volume of the eYFP^+^ cell population, a non-cell specific, general increase in mitosis was suggested (66). In analogy, a general increase in proliferating AP cells toward the end of pregnancy was also observed by Zhu et al. (31), with SOX2^+^ stem cells remaining mostly quiescent (31). On the other hand, estradiol, a regulator of PRL production during lactation, induces a small, 10% increase in dividing SOX2^+^ cells (6).

A novel and powerful technology to unravel tissue cell landscape and dynamics is provided by single-cell RNA-sequencing (scRNA-seq). In a recent study interrogating the pituitary of 13-week old lactating mice and age-matched virgin control animals (67), expansion of the lactotrope cluster was observed in the lactating mice (36% as compared to 29% in control), also showing upregulated expression of the neuroendocrine vesicle secretory protein chromogranin B (*Chgb*). Upregulation of PRL levels during lactation thus may reflect the combined effect of an expanded lactotrope population and a more active PRL-secretory machinery. Authors further speculated that the newly formed lactotropes are derived from multi-hormonal cells, since the latter cluster is reduced in abundance during lactation (10% as compared to 13% in control), expression of *Prl* within this remaining multi-hormonal cluster significantly upregulated and expression of other hormone transcripts (e.g., *Lhb)* downregulated. In addition, transdifferentiation of somatotropes to lactotropes was also proposed to play a role since *Prl* expression was clearly higher in the somatotrope cluster of lactating animals. Importantly, whether stem cells are involved in the expansion of the lactotrope population was not looked at or speculated on. Interestingly, the stem cell cluster appeared to become smaller in the lactating animals [3.2% as compared to 4.5% in control (67)].

After weaning of the progeny, lactotrope cell number returns to normal steady-state levels, supposedly through a combination of apoptosis and transdifferentiation away from the lactotrope phenotype. However, using the PRL^eYFP/+^ reporter mouse line, transdifferentiation was only observed at very limited rate [i.e., GH^+^ cells derived from lactotropes did not exceed 1% (66)].

Taken together, current data do not point to a significant role for stem cells in the generation of new lactotropes during pregnancy and lactation, but rather endow the committed progenitor cells with this function. Either, further and more detailed stem cell lineage tracing may indicate otherwise, or stem cells may rather play a nutritive, paracrine regulatory, or stimulatory role (**Figure 1**).

### Pituitary Stem Cells During Aging

Aging is associated with a deterioration in tissue homeostatic turnover and reparative capacity. In some tissues such as muscle, this waned behavior is causally associated with decreased stem cell number and functionality (68–71). Manifestations of aging are also observed in the endocrine system, including in pituitary output. Higher baseline TSH levels and altered ACTH dynamics have been observed in the aged population (18). In rat, thyrotrope cell number decreases with age, but TSH^+^ cell area (volume density) and TSH serum levels increase (72), consistent with observations in humans (18). These findings suggest a desensitization of the pituitary-thyroid axis during aging. Gonadotropes also show aging-associated changes. In the male rat pituitary, gonadotrope cell number and size decrease with a phenotypic shift from large to small vesicles in the cells, the latter being the main type present in the female gland (73, 74). FSH and LH levels decrease with aging in male rats, whereas they remain constant or even rise in female rats. Signs of degeneration (such as pyknosis) are observed in the aging gland gonadotropes (73). One of the best known aging-associated endocrine events is the so-called somatopause, a progressive fall in basal GH levels and resultant circulating IGF1 (75). Especially the GH pulse amplitude, but not the frequency, is reduced in aged individuals (18). In rats, number of GH^+^ cells was not found different between young (3-month old) and old (24-month old) animals (76). By contrast, in mice and humans GH^+^ cell number decreases at aging (77, 78). In addition to these putative local pituitary causes of dropped GH levels, also regulators of GH production alter when getting older, *viz.* dropped levels of activators such as ghrelin and GHRH and rising concentrations of inhibitors such as somatostatin. Furthermore, changed sensitivity to these regulators may play a role including a gender-independent decline in GH cell responsiveness to GHRH and ghrelin (79, 80).

Although involvement of pituitary stem cells in the gland’s aging phenotype has been regularly hypothesized (2, 21), this assumption is at present only scarcely supported. FS cells, known to encompass pituitary stem cells, are decreased in number in old rats [from 4.5 cells per reference area in young rats to 1.5 in old rats (81)]. However, in human pituitary, FS cell number was found to be expanded (82), an increase which might be grounded in the stem cell fraction of the heterogeneous FS cell population, but also in the stromal or immune subpopulations. In other tissues like muscle and heart, it has been observed that the stem cell population is negatively affected by age, presenting as a decline in number and in regenerative capacity (68–71). Interestingly, restorative capacity of the pituitary also fades at aging, in a quite speedily manner. Following infliction of injury in the gland of middle-aged (8- to 10-month old) mice using the GHCre/iDTR model (see below), regeneration—which plainly unfolds in young 8–12-week old animals—does not occur any longer. This regenerative failure in older mice coincides with a decline in pituitary stem cell number and fitness [i.e., decreased sphere formation capacity (21)].

Taken together, several changes occur in the pituitary’s cell landscape and hormonal output during aging, but at present, barely anything is known about the impact of aging on the local stem cells and their participation in these changes. The least that is known is that their number, activity and fitness appear to decline, as occurring with stem cells in many other tissues (21, 68–71).

## Pituitary Stem Cells During Physio-Pathological Conditions

### Pituitary Stem Cells During Stress

Stress is a physiological coping mechanism marked by activation of the hypothalamic-pituitary-adrenal (HPA) axis and essentially resulting in increased levels of circulating ACTH and glucocorticoids (cortisol in humans and corticosterone in mice). Other pituitary hormones are also affected by stress with impact depending on severity and exposure time (13, 83).

In mice, acute (30 min) cold stress does not result in immediate changes of corticotrope cell number, although corticosterone levels rise (14). The relative abundance of the other AP cell types is increased at the expense of non-hormonal cells. Since these changes occur acutely after application of the stressor, it was proposed that the new endocrine cells develop through maturation of committed progenitor cells that already transcribe or translate minimal (undetectable) amounts of hormone and rapidly increase this expression upon stress. In contrast, a 30 min cold exposure in rats was found to result in an increase in POMC^+^ and ACTH^+^ cell number and size (15). Another study also showed an expansion of cells expressing *Pomc* mRNA (as analyzed by *in situ* hybridization) 2–3 h after applying a cold stressor in rats (84). Exposure to chemical stressors (such as formaldehyde) also results in a rise of ACTH^+^ cell proportion as analyzed in mice (85). Following exposure of rats to increased ambient temperature, ACTH^+^ cells show reduced volume density, likely due to elevated secretion of ACTH from the corticotrope cells, which is concordant with the higher levels of ACTH (and corticosterone) found in the circulation (86).

Apart from the corticotropes as logical stress target, also other pituitary hormonal cells are affected. Thyrotropes respond to acute cold in rats by expanding in number and size (15). Somatotrope dynamics change depending on the type of stressor. Pituitary GH protein levels in rats increase upon acute immobilization or restraint but not after repeated immobilization. However, size and density of GH^+^ cells remain largely comparable to control (87). Prenatal dexamethasone treatment in rats, resulting in intensified HPA activity in postnatal life—and thus a chronically elevated stress state—triggered long-term changes in FS cell morphology but not number, whereas not affecting the corticotrope population (88).

In the HPA axis, the adrenal gland negatively feeds back through corticoids to the hypothalamus and pituitary, thereby keeping ACTH production under control. Hence, adrenalectomy causes a rise in ACTH level and a transient increase in ACTH^+^ cells in the pituitary (33). Mechanisms underlying this corticotrope remodeling include increased proliferation of existing ACTH^+^ cells which thus re-enter the cell cycle [4.5% dividing ACTH^+^ cells per unit area after adrenalectomy as compared to 0.5% in control (89)] and development of new corticotropes from stem cells, as demonstrated by SOX9 lineage tracing [i.e., 20% of the new ACTH^+^ cells originate from the traced cells (6)]. Another study showed an increase in proliferating TBX19^+^ corticotrope progenitor cells after adrenalectomy (90).

Overall, although pituitary cell remodeling during or following stress has been studied in different models, not much is known yet on whether and how pituitary stem cells play a role (**Figure 1**).

### Pituitary Stem Cells During Tumorigenesis in the Gland

Tumors in the pituitary gland, nowadays referred to as pituitary neuroendocrine tumors (PitNETs), represent 15% of all intracranial lesions and occur as symptom-causing tumors with a prevalence of 1 in 1,000 (1, 91). PitNETs can cause serious morbidity through hormone hypersecretion or, at the other side of the spectrum, reduced pituitary function (hypopituitarism) because of compressing healthy, neighboring pituitary tissue (1, 91). Not much is known on the underlying pathogenesis of PitNETs and their link with pituitary stem cells has so far only scarcely been studied. In search for so-called tumor stem cells (TSC), defined as cells driving tumor initiation, growth and heterogenic composition (thus displaying the orthodox stem cell properties of self-renewal and multipotency), and responsible for tumor re-growth after therapy because of their increased resistance, a SP was found in human and mouse PitNETs showing tumorsphere formation, expression of stemness markers and *in vivo* growth capacity, all pointing to a TSC character (92). Also other studies provided data proposing the existence of TSC in pituitary tumors [extensively reviewed in (93)]. Using a mouse model of lactotrope tumor (prolactinoma) formation in the pituitary (i.e., *Drd2* ^-/-^ mice), an increase in SOX2^+^ cells was found in the tumorous gland, at least partly due to elevated proliferative activity (92). These findings suggest that the resident pituitary stem cells are activated in case of the “threatening” tumorigenic event in the gland. Whether this reaction is preventing fierce tumor growth progression, or is otherwise stimulating the process, is not known. Moreover, the SOX2^+^ cells may not only represent activated tissue stem cells but also TSC developed from the(se) stem cells (or alternatively from other cell types by, for instance, dedifferentiation). However, direct descent of tumor cells from SOX2^+^ cells was not supported in the *Drd2* ^-/-^ mouse using SOX2^+^ lineage tracing (93), thus questioning their role as TSC. Rather, paracrine stimulation of tumor formation and growth may occur (**Figure 1**), as has also been reported in another pituitary-located tumor model (94). Transgenic expression of a genetically mutated, constitutively active form of the WNT signaling transducer β-catenin in embryonic pituitary progenitor cells (95) or in SOX2^+^ cells (7) results in the development of lesions in the gland showing characteristics of adamantinomatous craniopharyngoma (ACP), a benign but burdening tumor mostly occurring in children (96). The tumors contained typical nucleocytoplasmic β-catenin^+^ cell foci expressing the stem cell markers SOX2 and NESTIN (7, 95). However, SOX2^+^ lineage tracing revealed that the tumor proper did not directly derive from the SOX2^+^ stem cells, which, on the other hand, were characterized to produce several factors that could fuel tumor development and growth from the neighboring cells (7, 94–96). Intriguingly, tumors did not develop when β-catenin was transgenically expressed in committed (PIT1^+^) or differentiated (GH^+^, PRL^+^) cells, indicating an essential (although indirect) role of stem cells in this tumorigenic process (95). In accordance with the aberrantly activated WNT pathway in the stem/progenitor cells of this ACP-resembling model, upregulated expression of WNT components has also been observed in the SP of human PitNETs (92). Other typical stem cell-regulating pathways that have been advanced to potentially play a role in pituitary tumorigenesis are the NOTCH pathway, which seems either activated or suppressed in the tumor (analyzed as a whole), depending on the pituitary tumor subtype (97), and the HIPPO pathway. Elevated expression of HIPPO pathway components (e.g., *YAP1*, *LATS2*) was detected in the SP of PitNETs (92). Moreover, genetically induced elevation of YAP/TAZ signaling in the SOX2^+^ pituitary stem cells results in the development of non-secreting aggressive tumors (98). Intriguingly, in contrast to the *Drd2* ^-/-^ prolactinoma and ACP-resembling models described above, these tumors directly (and clonally) originated from deregulated SOX2^+^ cells as investigated using SOX2^+^ lineage tracing (98). However, these highly proliferative carcinoma-resembling tumors do not immediately draw a parallel with the typically benign tumors that occur in the pituitary. Finally, a recent study, clustering pituitary tumors by RNA-seq analysis in three groups coinciding with canonical lineage transcription factors [i.e., TBX19, NR5A1 (SF1) and POU1F1 (PIT1)], did not reveal a transcriptomic link with the (normal) stem cell population, but suggested that the “tumor progenitor cells” (TSC) derive from already (partially) committed cells expressing the respective transcription factor (99).

Taken together, although more and more studied, no clear view is shed yet on the connection between pituitary stem cells and tumorigenesis in the gland. It remains essential to decipher whether and how pituitary stem cells are implicated in order to advance our knowledge on pituitary tumorigenesis, at present only poorly understood. Of note, the local stem cells may become “activated” during tumorigenesis not only by genetic or molecular (signaling/growth factor) aberrations, but also by the accompanying physical tissue damage, a reaction indeed observed following inflicted injury in the gland.

### Pituitary Stem Cells Following Injury in the Gland

Hypopituitarism, involving deficiency in one or more pituitary hormones, results in serious morbidity given the gland’s central position in the endocrine system (100). This hypofunction may be due to faults in embryonic development, or may be caused by damage occurring during postnatal life. Culprits include hemorrhagic necrosis in the gland (Sheegan’s syndrome), physical damage by tumor growth as well as following its operational resection, and destructive impact on the gland through head trauma [traumatic brain injury (TBI)] as caused by traffic, sport or violence accidents. To define the behavior of the gland’s stem cells following injury, a transgenic mouse model was created allowing the destruction of pituitary cells (19). Before, partial hypophysectomy had been applied which is technically challenging and difficult to standardize. An older study in rats reported an increase in “chromophobic” cells in the residual pituitary after partial tissue removal, which may represent the immature (stem) cells (22, 101). However, no cell regeneration or anatomical restoration was observed, even up to one year later (101). In the more recent transgenic pituitary injury model, specific cell populations are targeted for killing by diphtheria toxin (DT) treatment. In the GHCre/iDTR mouse model, expression of the DT receptor (DTR) is induced (hence, “inducible” or iDTR) through the activity of Cre recombinase, expressed under control of the GH promoter. DTR in the GH-expressing cells then makes these cells sensitive to DT-induced ablation. A 3-day DT injection results in major (80–90%) obliteration of the somatotrope cells, thereby realizing a controlled pituitary injury model (19). Interestingly, the resident stem cells promptly react to the cell-ablation damage in the gland. First, it was found that the stem cell population shows augmented proliferative activity and expands (about 2-fold) upon injury based on several read-outs including SP, FS cell and SOX2^+^ phenotype and sphere-initiating capacity (19). Morphologically, especially the wedge regions show a prominent enlargement of the SOX2^+^ stem cell zone (19). In addition, upregulated gene expression of factors typically associated with stem/progenitor cells and/or with pituitary embryogenesis (such as FGF, BMP, and components of the SHH, WNT, and NOTCH signaling pathways), further supported the activated status of the pituitary stem cell pool following injury. Similar facets of stem cell activation were observed in a complementary pituitary injury model killing the PRL-expressing cells (with 70% ablation grade) using the PRLCre/iDTR mouse model (20). Finally, a proliferative activation reaction was also detected in the remaining SOX2^+^ cell compartment after DT-targeted SOX2^+^ cell ablation (46). However, this response only unfolds in early-postnatal mice (1–4 weeks of age), not in adult animals (8–12 weeks), and does not result in restoration of SOX2^+^ cell numbers as analyzed 4–6 months later (see above).

Interestingly, a significant restoration of the ablated cell population was noticed, thereby convincingly demonstrating for the first time that the adult pituitary gland possesses regenerative capacity. The number of somatotropes is restored to 50–60% after 4–5 months [GHCre/iDTR model (19)]. Also GH serum levels substantially re-lift [to 30% (21)]. However, regeneration does not reach higher levels, even following an extended follow-up period (19 months) after the somatotrope ablation insult, suggesting that full regeneration is not critical for survival and supportable life (21). Since the stem cell compartment is activated upon injury followed by tissue repair, the question presented whether stem cells are involved in the regenerative response. Co-expression of SOX2 and GH surfaces in several cells, not observed in the normal steady-state gland, thereby pointing to differentiation of (reacting) stem cells toward the somatotrope fate. No evidence was found for contribution of other mechanisms such as transdifferentiation from lactotropes (no increase in PRL^+^/GH^+^ cells) or proliferation of remaining somatotropes [dividing GH^+^ cells are virtually non-existent (19)] (**Figure 1**). Nevertheless, direct demonstration of the descent of the newborn GH^+^ cells from the SOX2^+^ stem cells awaits lineage tracing, however at present technically difficult using a Cre-mediated approach. For instance, tamoxifen-induced SOX2CreERT-driven lineage tracing after GHCre-mediated somatotrope ablation cannot exclude simultaneous tracing of the remaining GH cells (also expressing Cre) and will thus not allow to discern between reporter^+^ cells derived from GH^+^ or SOX2^+^ cells during the regenerative period. As an alternative mechanism underlying regeneration, the activated stem cells may act as restoration-stimulating signaling center, for instance driving the PIT1^+^ progenitor cells into proliferation [as apparently also occurring in pregnancy/lactation and probably also during neonatal maturation, as discussed above (31)] (**Figure 1**). Involvement of stem cells in the regenerative reaction, whatever the mechanism, is further supported by upregulated gene expression in the stem cells of factors belonging to pathways typically involved in tissue regeneration such as the epidermal growth factor (EGF), FGF, EMT, and Hippo signaling systems (21), and of factors playing a role in pituitary embryogenesis (see above), suggesting recapitulation of the embryonic developmental program, as also reported in other regenerating tissues such as muscle (102). In the PRLCre/iDTR model, the lactotrope population shows a swifter restoration, reaching 60–70% after 6–8 weeks (20). The more efficient repair is likely due to the accumulated action of multiple regenerative processes in this cell lineage, including stem cell differentiation (increase in SOX2^+^/PRL^+^ co-expressing cells), amplification in lactotrope proliferation (as observed) and transdifferentiation of somatotropes to lactotropes [increase in cells co-expressing GH and PRL (20)] (**Figure 1**). Formerly, some signs of regeneration were also briefly reported following thymidine kinase-mediated obliteration of somatotropes and lactotropes (103). However, underlying mechanisms, and particularly the involvement of stem cells, were not investigated at that time. Moreover, this nucleotide (FIAU)-incorporating approach only kills cells that are actively dividing and therefore necessitated FIAU treatment from the embryonic till early-postnatal stage (103), thus excluding applicability of the technique for cell ablation and regeneration studies in the predominantly non-mitogenic adult pituitary.

TBI is increasingly recognized as an important cause of hypopituitarism with drop in hormone levels (especially GH and ACTH), possibly due to damage to the pituitary tissue, either directly or through hypothalamic or vascular impacts (104, 105). It has been found that hormone serum levels in TBI patients may restore after several (3 to 36) months (104, 106–108). Whether this recovery is due to compensatory behavior of the remaining pituitary cells or the hypothalamus-pituitary axis, to regained pituitary cell functionality, or to repair of the pituitary insult with new cell formation to replace damaged and destroyed cells, is not known (104, 105). Further in-depth investigation of the pituitary cell landscape upon TBI, for instance using scRNA-seq analysis, is expected to provide deeper insight into (stem) cell reaction and possible repair, and into TBI-induced hypopituitarism, which may eventually lead to new approaches to clinically deal with this prevalent endocrine deficiency condition.

Taken together, the resident stem cell compartment of the pituitary promptly reacts to injury in the gland, which may lead to ensuing regenerative processes. However, underlying mechanisms, whether it involves direct generation of new cells or indirect paracrine stimulation of cell neogenesis, still need to be clearly defined (**Figure 1**). In the longer run, this knowledge may pave the way to regenerative therapies in case of damage-induced hypopituitarism such as by tumor operation or head trauma. Pituitary stem cells could potentially be stimulated *in vivo* to drive new-formation of destroyed endocrine cell type(s), or may *ex vivo* be expanded, differentiated and transplanted, as proposed already a long time ago in a study in which chromophobes (freshly isolated from the pituitary) were shown to divide and differentiate when transplanted into the hypophysiotropic area of the rat (109). Alternatively, the secretome of activated pituitary stem cells, when functioning as signaling centers, may be defined and important factors applied as treatment *in vivo* (22) (**Figure 1**). As an important remark, it is still unclear whether also the human pituitary is capable of regenerating. Only very few studies addressed this issue. Following transsphenoidal electrocoagulation-mediated destruction of metastasized tumors in the gland (thus damaging the pituitary tissue), actively dividing chromophobic cells around the necrotic core and signs of glandular regeneration were observed in post-mortem pituitaries (110). As described above, restoration of pituitary function in terms of circulating hormone levels after TBI might also involve pituitary regeneration although at present not investigated. Clinical endurance of hypopituitarism, when brought about by local tumor growth or removal, does not immediately point to a restorative response. However, transient regenerative processes may occur which are stalled for one or the other reason, but which cannot be probed in human beings and will need innovative *in vitro* approaches.

## *In Vitro* Model Systems to Study Pituitary Stem Cells and Remodeling

Although studies in experimental animals, in particular mice, have generated important information on pituitary development and functioning, there is a high need for appropriate *in vitro* research models to study pituitary stem cell biology and pituitary (cell) remodeling, and their cross-section. Over the years, several different pituitary *in vitro* models have been developed, ranging from 2D immortalized cell lines to 3D structures, each with own specific sets of advantages and shortcomings (**Table 1**). Here, we give a short overview of the different model systems and their potential as research tool to untangle pituitary remodeling and in particular the biology, role and regulation of the pituitary stem cells in these plastic adaptations.

**Table 1:** Pros and cons of *in vitro* pituitary study models.

Model	Pros	Cons
***Pituitary cell lines***	- Readily available - Easy to handle and manipulate	- Tumor-derived or genetically immortalized (not representative for normal tissue cells) - Only represents a single cell type - 2D format
**From primary compound pituitary**		
***Monolayer cell culture***	- More pituitary cell types - Easily established	- Limited expandability - Quickly lose physiological behavior - 2D format
***Explant culture***	- Representative (reflects tissue heterogeneity and physiological responses) - Easily established - 3D format	- Limited expandability – necrosis - Limited experimental possibilities or manipulations
***Cell aggregate culture***	- Representative (reflects tissue heterogeneity and physiological responses) - Easily established - Long-term culture - 3D format	- Limited expandability - Difficult to enrich for FS/stem cells
**From primary pituitary stem cells**		
***Sphere culture***	- Quite representative (differentiation into hormonal cells) - Allows exploration of pituitary stem cell biology - 3D format	- Limited expandability - Limited application potential (e.g., to unravel differentiation processes)
***Colony culture***	- Quite representative (differentiation into hormonal cells) - Allows exploration of pituitary stem cell biology	- Limited expandability - Limited application potential (e.g., to unravel differentiation processes) - 2D format
***Organoid culture***	- Extensive expandability of limited primary (stem) cells - Quite representative (differentiation into specific hormonal cells) - Allows exploration of pituitary stem cell biology - High application potential (e.g., tumor/disease modeling, drug screening, …) - Amenable to gene editing - Cryopreservable - 3D format	- Cost-intensive - At present still limited differentiation - Very hard to achieve from normal human pituitary
**From PSCs**		
***Organoid culture***	- More representative (may reflect tissue heterogeneity) - ESCs/iPSCs are readily available and expandable - Model for human normal pituitary - 3D format	- Labor intensive set-up - Cost-intensive - Limited expandability once differentiated into pituitary fate - Human iPSCs known difficult to transfect
		

### Pituitary Cell Lines

Cell lines have been developed that represent different pituitary cell types. The lines were derived by culturing pituitary tumors or by genetically transforming and immortalizing pituitary cells or specific cell types. Well-known examples are the mouse corticotrope AtT20 cell line and the rat lactosomatotrope GH3 cells. The cell lines proved valuable to study hormone regulation by hypothalamic and other factors (111, 112). FS cell lines have also been established [e.g., PDFS (113)] and have been instrumental to study cytokine regulation of AP cell functions (114, 115) and intrapituitary communication (111, 116).

Despite their value, cell lines have important shortcomings in mimicking pituitary (cell) physiology, in particular because of their transformed pheno-/genotype (usually aneuploid and neoplastic), their 2D culture format (contrasting with the 3D configuration and anchoring *in vivo*), and their poor or non-existing representation of the compound pituitary cell composition. On the other hand, the cell lines still appear to contain a (driving)? progenitor cell population. For instance, SP cells were identified in AtT20 and GH3 cells showing stemness functionality such as sphere formation and xenotransplant tumor growth (92).

### Model Systems Established From Primary Pituitary

#### Models Starting From the Compound Pituitary

Primary cells from dissociated pituitary glands can be cultured as monolayers, either as original mixture or following enrichment of specific cell types using sedimentation gradients (117, 118) or counterflow centrifugation (119). Although easy to achieve, the resultant 2D cultured cells quickly (within one week) start to deteriorate, lose their normal physiological behavior and responsiveness (e.g., to hypothalamic hormones), and die.

Alternatively, primary pituitary tissue can be kept in culture as explants, frequently employed in the past (49, 120, 121). To do so, pituitary tissue fragments (e.g., halved glands) are cultured either submerged in optimized culture medium (composition differing between studies), or deposited at the air-liquid interface [e.g., RP explants (29, 122, 123)]. Although physiological responses are elicited in explant cultures, their “shelf life” is limited because of inadequate perfusion, lack of sufficient oxygenation and resulting necrosis in the core (after a few days in culture).

Above-mentioned shortcomings are overcome in an organotypic culture system of pituitary cell aggregates (44, 48, 124, 125). Dissociated primary pituitary cells (typically from rat or mouse) are cultured under constant gyratory movement in a pituitary-optimized, serum-free defined medium (SFDM), allowing the cells to re-aggregate and form histotypic cultures containing all main pituitary cell types [including FS and stem cells but lacking primary pituitary endothelial cells and housed lymphocytes and monocytes (48)], thereby reflecting the major pituitary cell composition as well as cell type-typical organization. Pituitary cell (re-)aggregates can be kept in culture for months without losing tissue and cell-type representation and functionality (i.e., hormone production capacity and regulation with responsiveness to hypothalamic hormones). As true in the *in situ* gland, not much cell proliferation is occurring in the aggregates, and cultures are not expanding but static in abundance. Although aggregates can be formed from enriched endocrine cell types, no aggregates have been established from isolated stem cells, and aggregates from enriched FS cells remain small and fragile (44, 48, 124, 125).

#### Non-Organoid Models Derived From Pituitary Stem Cells

A common and essential shortcoming of the above-mentioned pituitary model systems is that at present none of them allows to specifically grow and study the stem cell compartment. *In vitro* study models for pituitary stem cells are essential to advance our knowledge on the behavior (self-renewal, multipotent differentiation, activation), regulation and translational and clinical potential of this still under-defined pituitary cell population.

Clonal sphere formation is a general characteristic of stem cells, which was first defined for neural stem cells (126). Cultured in a specific growth medium including B27, EGF and/or basic FGF (bFGF/FGF2), stem cells clonally expand to form floating 3D spheres that can be serially passaged, based on the capacity of the sphere-initiating stem cell to self-renew and generate proliferative progenitor cells. Under specific culture conditions, differentiation to various cell types of the tissue of origin occurs in the spheres because of the (multipotent) differentiation capacity of the originating stem cell (126). Free-floating spheres can be derived from pituitary by culturing dissociated AP cells in SFDM (or other culture medium) supplemented with B27 and bFGF. These so-called pituispheres are composed of SOX2^+^ stem cells [also expressing other pituitary stem cell markers such as NESTIN, E-cadherin, S100 and SOX9 (3–5)], originate from the SP [and not from the non-SP (3)] and from the SOX2^+^ stem cells (6), and can be differentiated toward all pituitary hormonal cell types by culturing on an ECM (Matrigel)-coated surface in medium without stem cell growth factors. Pituispheres (when formed after 6 days of culture) can be dissociated and resultant cells re-seeded to grow new spheres, although the propagation efficiency gradually but quickly declines, being at present limited to 3–4 serial passages (3–5, 40). This stem cell model has proven its merits, mainly as read-out of stem cell functionality and activation (3–6, 16, 19, 31, 46, 48). For instance, more spheres are formed when starting from the damaged GHCre/iDTR pituitary, in line with the activated status of the stem cells upon injury [see above (19, 21)].

Another general test to probe stem cell phenotype and functionality is provided by the colony-forming assay, which, in contrast to the sphere assay, occurs in 2D format. Dissociated pituitary cells, cultured in medium containing serum, bFGF and cholera toxin, generate colonies after 6 days that express the stem cell markers NESTIN and SOX2, as well as limited levels of early pituitary development markers (LHX3), the latter increasing toward 14 days in culture (coinciding with the exit of the exponential growth phase in the colony). Eventually, the colonies contain cells of all five AP cell lineages, although at modest numbers (7, 96, 127).

Although valuable for specific readouts of stem cell functionality (probed by, for instance, colony-forming efficiency), the 2D format does not mimic the 3D anchoring and interaction of cells as present *in vivo*. Despite a 3D configuration, pituispheres remain limited in growth and application potential. Hence, a more versatile and efficient model system would definitely be instrumental to study pituitary and stem-cell biology *in vitro*. Over the last years, so-called organoid models have been developed, either derived from pluripotent stem cells (PSCs) or from pituitary stem cells.

### Organoid Models Established From Pluripotent and Pituitary Stem Cells

In their contemporary meaning, organoids represent composite cell configurations that *in vitro* grow from (single) stem cells that self-renew, proliferate and self-organize in 3D, eventually replicating key biological properties of the organ of origin (128, 129). Such present-day organoid modeling has been achieved starting from tissue stem cells and from PSCs.

Tissue stem cell-derived “organoid-ing” typically follows the principles that were laid down in the first successful development of such organoids (130). Tissue fragments or dissociated cells (encompassing the resident stem cells) are embedded in an ECM/basement membrane-mimicking 3D scaffold [such as Matrigel or basement membrane extract (BME)] and cultured in a cocktail of compounds encompassing generic stem cell regulators as well as factors operational in the stem cell niche of the specific tissue. The core of this blend is formed by EGF, Noggin and WNT activators. EGF is a potent mitogen that stimulates epithelial stem cell proliferation while Noggin acts as an inhibitor of BMP signaling which blocks stem cell differentiation. The WNT pathway is a well-known regulator of tissue stem cells, which in organoid culture is generally boosted by adding R-spondin 1 (RSPO1), a ligand of the leucine-rich repeat containing G protein-coupled receptor 5 (LGR5) which marks stem cells in multiple tissues, and which amplifies WNT signaling strength (128, 129, 131). Using this protocol, organoid models have meanwhile been developed from manifold tissues of both mouse and human origin such as stomach (132), liver (133), and endometrium (134, 135). A key asset of tissue stem cell-derived organoids is that they display strong, long-term expandability while robustly retaining their properties and remaining genomically stable (128, 129).

PSC-derived organoid models are achieved by mimicking the sequential steps that occur during embryogenesis of the specific tissue or organ. Embryonic stem cells (ESCs) and induced PSCs (iPSCs), either in 2D or 3D format, are treated with activators or inhibitors of specific embryogenic pathways in a consecutive manner. As an example, organoids mimicking the intestine were obtained by driving human PSC aggregates first toward definitive endoderm using activin A, and then toward hindgut endoderm using WNT3A and FGF4. The obtained floating spheroids were then embedded in Matrigel containing RSPO1, EGF, and Noggin to finally generate an intestinal organoid structure (136). Organoid modeling using PSCs is especially valuable for complex organs composed of several different structures or compartments and therefore difficult to sculpt using the tissue stem cells (which, moreover, are not always clearly identified for those organs), such as brain (137), liver (138), and kidney (139).

In general, organoids provide interesting and powerful application potential in both basic and translational research (**Table 1**). First, they represent a valuable tool to disentangle tissue development including stem cell biology. In addition, organoids can be used to model diseases (128, 129). For example, tumor tissue-derived organoids have been established from different sorts of cancer such as colorectal (140), breast (141), gastric (142), bladder (143), and endometrial cancers (135), resulting in large “living” (cryopreserved) organoid biobanks of patient-derived samples. This resource is highly instrumental for drug screening, which can give insight into inter-individual drug responses and may be translated into personalized medicine. Furthermore, organoids can be applied for studying infectious diseases. Eye-catching examples are the application of brain organoids to decipher Zika virus infection (144) and the use of blood vessel, kidney and intestinal organoids to probe and unravel the currently raging SARS-CoV2 infestation (145, 146). In the field of regenerative medicine, organoids may also prove constructive to restore damaged or diseased tissue. For instance, it has been shown that human colon-derived organoids move to experimentally damaged areas in mouse colon where they implant and form new tissue with self-renewing crypts that are histologically and functionally normal, thereby displaying long-term engraftment (147). As another example, organoids from primary human hepatocytes engraft and proliferate extensively when transplanted in damaged mouse liver (148).

Both organoid model systems, whether tissue stem cell- or PCS-derived, have pros and cons (**Table 1**). Tissue stem cell-derived organoids are important models to study postnatal tissue stem cell biology (phenotype, regulation, function, activation) which is less straightforward in PSC-derived organoid models in which the adult stem cell phenotype is (as yet) not, or not fully, recapitulated, but instead embryonic progenitor cells are (and can be more effectively studied). Tissue stem cell-derived organoids are long-term and exponentially expandable, allowing to multiply minute tissue samples (e.g., human biopsies) for extensive downstream applications. In contrast, PSC-derived organoids represent an end-point situation, not being passageable, although the starting material (PSCs) can be infinitely expanded first, and large numbers of organoids can then be developed. As already mentioned, PSC-derived organoids are more apt to reproduce complex organs, and different tissue cell types are co-formed during the “organoid-ing”, whereas adult stem cell-derived organoids solely reflect the tissue epithelial compartment. On the other hand, the latter reductionist model may be an asset in specific applications (such as simplified high-throughput screenings). Currently, strong efforts are being made to further advance the adult stem cell-derived organoid model by co-culturing epithelial (stem) cells with other cells present in the tissue microenvironment such as mesenchymal, endothelial and immune cells (149, 150). PSC-derived organoid models, by their very nature, more closely recapitulate the embryogenic path of the organ, thus providing simultaneous development of different intrinsic tissue-specific cell types, and in some cases (as described below for the pituitary) also extrinsic elements, i.e., neighboring structures that in real life co-develop with the tissue. Finally, both tissue stem cell- and PSC-derived organoids are amenable to gene editing (by, for instance, CRISPR/Cas9), although human iPSCs may show resistance to efficient transfection (151, 152).

#### Pituitary Organoids Derived From Pluripotent Stem Cells

About 10 years ago, pituitary organoid development was achieved starting from PSCs (153). Based on knowledge of pituitary embryogenesis, mouse ESCs, brought in an aggregate formation, were first directed to oral and neural ectoderm in adjacent layers using a chemically defined medium lacking any growth factors. The aggregates started to express the hypothalamic markers *Rax* and *Sox1* in the inner layer. As occurring *in vivo* where an intimate, contact-dependent interplay is needed between oral and neural (prospective hypothalamus) ectoderm for pituitary embryogenesis, co-existence of both layers in the 3D ESC configuration was essential to eventually result in the formation of a RP-like structure. This process presented as a morphological invagination and expression of the early pituitary transcription factors PITX1 and LHX3 at the border of both layers upon SHH activation, thereby repeating what is occurring *in vivo*. Further development toward different pituitary cell lineages was then reached also based on knowledge of mouse pituitary embryogenesis. Corticotropes (visualized by *Tbx19* and ACTH expression) were obtained by NOTCH inhibition using DAPT. Driving cells into the somatotrope and lactotrope fate was achieved following WNT pathway stimulation and subsequent exposure to glucocorticoids (for somatotropes) or estradiol (for lactotropes). Functionality was shown for corticotropes which were most efficiently derived (approximately 35% of the non-neural cells within the ESC-derived structure, meaning ∼3% of the total cell number). The differentiated ESC-derived aggregates responded *in vitro* to the hypothalamic releasing hormone CRH by increased ACTH release, and rescued *in vivo* ACTH levels and mouse lethality when subrenally transplanted into hypophysectomized mice (153). A few years later, this tour de force was repeated with *human* ESCs (154). Hormone-producing cells were generated with corticotropes developing spontaneously (approximately 12% of the PITX1^+^ non-neural cells), somatotropes (and to a lesser extent lactotropes and thyrotropes) appearing after glucocorticoid exposure, and LH- and FSH-expressing gonadotropes arising following NOTCH inhibition. Thus, specific differentiation was found to be different in mouse and human cell cultures (see e.g., the effect of NOTCH inhibition), highlighting important caveats when translating mouse developmental principles to humans. Cells responded to hypothalamic releasing hormones and increased human ACTH expression was observed when aggregate structures were grafted subrenally in hypophysectomized mice (154).

A few years earlier, pituitary hormonal cells were also generated from human ESCs as well as their somatic cell-derived counterparts (iPSCs), although in a 2D format (155 **)**. Here as well, SHH signaling was crucial for pituitary specification. ACTH^+^, GH^+^ and FSH^+^ hormonal cell types could be developed, with induction of corticotropes again found most efficient. NOTCH inhibition was necessary to increase the development of *PIT1-* and *GATA2*-expressing (gonadotrope) lineages. After subcutaneous grafting *in vivo* in immunodeficient mice, the differentiated cells showed long-term survival and production of human ACTH and GH. In a further refinement, dorsal-ventral patterning (as occurring in pituitary development *in vivo)* was imposed on the pituitary-directed human PSCs *in vitro* (156). Treatment with high BMP2 concentration resulted in upregulation of *FSHB* and *LHB* expression and the detection of FSH^+^ cells (being ventral cell types in embryonic development), whereas high FGF8 exposure yielded increased *POMC* expression and POMC^+^ cells (dorsal cell type in development), both findings in accordance with knowledge on mouse pituitary embryogenesis in which an opposing BMP2-FGF8 signaling gradient determines dorsal-ventral patterning and regional cell type specification (157). Intermediate concentrations of both factors resulted in an increase in cells expressing *GH, PRL*, and *TSHB* as well as of GH^+^ cells, which are *in vivo* also found in the transitional zone during development (158). Subcutaneous injection of the differentiated cells (embedded in Matrigel) in hypophysectomized mice showed functional somatotrope and corticotrope cells producing (human) GH and ACTH, respectively (156).

Some recent studies further built on the 3D pituitary-developing, human ESC-based organoid model of Ozone et al (154). After transposing the technique to human iPSCs, further endocrine maturation was achieved through creating “hypothalamic-pituitary units” by refining the culture method, but especially by prolonging the culture period (up to 500 days) allowing the hypothalamic neurons to further mature, which concurrently resulted in more progressed development of the pituitary corticotropes (159). Indeed, the resultant units were found to yield better differentiation of ACTH^+^ cells with higher ACTH secretion ability (paralleling levels in adult mice), and were found functional, showing, for instance, physiological responses to hypoglycemic stress in releasing CRH and ACTH. One caveat, as reported, is that prominent results were only obtained with one iPSC line while three lines were tested showing wide variation in hypothalamus-pituitary induction efficiency (159). Interestingly, the pituitary-developing organoid model was also applied to study pathogenetic mechanisms underlying congenital pituitary hypoplasia (CPH), caused by mutations in orthodenticle homeobox 2 (*OTX2*), known to be important in the development of forebrain, eye and pituitary (160). *OTX2*-mutant CPH patient-derived iPSCs were subjected to the pituitary-developing organoid protocol which revealed weakened LHX3 expression, associated with increased apoptosis of the pituitary progenitor cells and impaired differentiation into AP. It was found that OTX2, expressed in the hypothalamic part of the 3D *in vitro* aggregate, regulates LHX3 expression in the oral ectoderm part *via* hypothalamic FGF10 expression, eventually essential for progenitor cell maintenance (160). The phenotype was rescued by correcting the mutation, whereas introducing the mutation in control iPSCs resulted in a similar apoptotic picture. Finally, Kanie et al (161). produced iPSCs from an anti-PIT1 syndrome (hypopituitarism) patient and control person which were subjected to the pituitary development protocol (161). Expression of LHX3 was observed after 40 days in culture and of GH, PIT1 and ACTH in some cells after 100 days. PIT1 was found to undergo antigenic processing and presentation; however, no difference was observed between patient and control model system. Together, these studies provide the first human pituitary organoid disease models, nicely illustrating the power and future direction in unraveling (genetic) human pituitary disease by using iPSC-derived organoid model systems.

Taken together, these novel PSC-derived research models provide important tools to study pituitary development which can especially advance our knowledge on *human* pituitary biology and embryogenesis (at present only concluded from snapshot or longitudinal imaging analyses of embryos or patients with aberrant pituitary development), and/or to generate pituitary endocrine cells for transplantation purposes, since PSCs are amenable to substantial expansion, and large numbers of pituitary endocrine cells can theoretically be generated (**Table 1**).

#### Organoids Derived From Pituitary Stem Cells

Very recently, organoids were established from pituitary stem cells (162). Thorough evaluation of the typical organoid growth factors (see above) and of multiple pituitary embryogenesis-related signaling molecules such as FGF8, FGF10, and SHH led to the definition of an optimized medium in which organoids could be developed from (mouse) adult AP. The organoids originate from the SOX2^+^ pituitary stem cells as shown by utilizing FACS-sorted SOX2^eGFP+^ cells [from the SOX2 reporter mouse model Sox2^eGFP/+^ (163)], whereas organoids do not form in cultures of the SOX2^eGFPneg^ cell fraction. The obtained organoids represent the stem cell compartment, as they are completely composed of cells expressing pituitary stem cell markers (such as SOX2 and E-cadherin). After passaging (i.e., breaking the organoids up into fragments which are reseeded in culture, thereby expanding them), the organoids retain this stemness phenotype. Upon transplanting the organoids under the mouse kidney capsule, they engraft and differentiate toward endocrine lineages, albeit at a modest extent (162). To study pituitary stem cell biology and activation after injury, the designed organoid method was applied to the GHCre/iDTR damage-and-regeneration mouse model [as described above (19)]. The efficiency of pituitary organoid formation was found to be higher when starting from the damaged (GHCre/iDTR) than the control, undamaged (-/iDTR) pituitary, in agreement with the activated state of the pituitary stem cells after the inflicted injury (162). Increase in organoid formation efficiency was also observed when starting from the neonatal gland (162), again recapitulating the boosted activation status of the stem cell compartment (see above). RNA-seq analysis of the organoids revealed novel pituitary stem cell labels (such as *Krt8* and *Krt18*) and damage-upregulated stem cell markers [such as *Prrx1/2*; see also (164)] which were all confirmed *in vivo*, illustrating the high potential of this new organoid model as pituitary stem cell biology research tool with reliable *in vivo* correlate and translatability (162). The model system can be applied to unravel pituitary stem cell biology and activation, or inhibition, during the several conditions of pituitary remodeling as reported above. Moreover, this particular organoid technology can also be used to study pituitary diseases, in particular tumors from which organoids could be developed, and utilized to explore tumorigenic mechanisms and impact of existing medicines, or new pipeline drugs.

Taken together, pituitary organoid models have been developed from PSCs and pituitary stem cells. Both organoid types represent interesting, complementary tools to study pituitary biology, each with own advantages and specific applications (**Table 1**). The PSC-derived model is most apt to study pituitary embryogenesis, moreover enabling the concomitant development of non-epithelial tissue cell types and of the neighoring hypothalamus. In addition, the model can be started from human PSCs, thereby providing a human *in vitro* pituitary model. The pituitary stem cell-derived organoid model is most suitable to study postnatal pituitary stem cell biology including in-depth characterization of phenotype and of regulatory and activating molecular networks. However, obtaining human normal pituitary tissue (e.g., from autopsy) is very hard to achieve. In contrast, human pituitary tumor samples can be readily obtained from surgery to be turned into tumor organoid models, while the PSC-derived technique is more apt to develop genetic disease models (as illustrated above). At present, the PSC-derived organoid model shows more efficient generation of hormone-producing cells, making it (currently) more apt for unraveling endocrine lineage development and maturation, and for potential regenerative purposes. Technically, setting up pituitary stem cell-derived organoids is less labor-intensive and achieved in a short period of time (within 10 days), whereas the PSC-derived model is more hands-on and takes a longer time to develop and mature (from 1 month to 500 days). Moreover, results should be confirmed in several different iPSC lines which are known to be considerably heterogenous in genomic make-up and biological behavior [see (159)].

## Conclusion

Plastic adaptation of its cell landscape to changing situations in life is an important property of the pituitary gland. Although these changes in cell constitution and hormonal output are known to happen, not much is understood yet on how they are brought about. Here, we looked at this question focusing on the stem cell position in this dynamic play. Although discovered more than a decade ago, many key questions regarding their regulation and biological function and significance remain today. Overall, their contribution to the dynamic cell adaptations as observed in the pituitary remains largely unresolved. With the emergence of new technologies including single-cell transcriptomics and pituitary organoid culturing, several of these questions can be tackled in the near future.

## Author Contributions

EL collected all the information and wrote the manuscript. AV provided input on the aging pituitary. HV co-wrote, critically revised, and finalized the manuscript. All authors contributed to the article and approved the submitted version.

## Funding

This work was supported by grants from the KU Leuven Research Fund and the Fund for Scientific Research (FWO) – Flanders (Belgium). EL and AV are supported by a PhD Fellowship from the FWO (11A3320N and 1141717N, respectivey).

## Conflict of Interest

The authors declare that the research was conducted in the absence of any commercial or financial relationships that could be construed as a potential conflict of interest.
